# The Italian Alpine and *Subalpine trouts*: Taxonomy, Evolution, and Conservation

**DOI:** 10.3390/biology11040576

**Published:** 2022-04-11

**Authors:** Gianluca Polgar, Mattia Iaia, Tommaso Righi, Pietro Volta

**Affiliations:** Water Research Institute (IRSA)—CNR, Largo Tonolli 50, 28922 Verbania Pallanza, Italy; mattia.iaia@irsa.cnr.it (M.I.); tommaso.righi@irsa.cnr.it (T.R.); pietro.volta@cnr.it (P.V.)

**Keywords:** recreational fisheries fishery management, introgressive hybridization, stocking, non-native species, allochthonous species, trout fishing, trout taxonomy

## Abstract

**Simple Summary:**

In a great part of the world, trout fishing has long inspired human spiritual ideals of immersion into nature and recreation, far removed from the fast-encroaching urbanization. Concurrently, these values and emotions fueled a white-hot business, establishing a florid market of outdoor recreation. Since the 20th century, the trout-culture industry strived to provide anglers with fishing entertainment by stocking massive amounts of non-native trouts in dozens of countries, irrespective of the lakes’ and rivers’ carrying capacity. This had dire consequences on the structural and functional diversity of these ecosystems. “Trout wars” sparked throughout the world between the promoters of stocking activities and the promoters of “wild trout management” and ethics. The “Italian trout war” has been fought on the harsh battleground of trout taxonomy, ecology, distribution, and native vs. non-native interfertile species. Northern Italy, home to the Italian Alpine and subalpine trouts and economic center of the national trout-culture and stocking industry, was particularly affected by this clash. We review here the state of art of this ongoing debate, outlining our scientific view of the taxonomy, evolution, distribution, and sustainable management of the native Italian trouts of northern Italy.

**Abstract:**

During the last 150 years, the trout-culture industry focused on enhancing trout populations by stocking, in response to the growing anglers’ demand and the habitat degradation associated to the rapid urbanization and hydropower development. The industrialized north of Italy, home to the Italian Alpine and subalpine trout populations, is the source of most of the revenues of the national trout-culture industry. Its rapid growth, and the massive introduction of non-native interfertile trouts eroded the genetic diversity of native lineages, leading to harsh confrontations between scientists, institutions, and sportfishing associations. We review here the state of the art of the taxonomy and distribution of the northern Italian native trouts, presenting both scientific results and historical documentation. We think the only native trouts in this region are *Salmo marmoratus*, widespread in this region, plus small and fragmented populations of *S. ghigii*, present only in the South-western Alps. We strongly recommend the interruption of stocking of domesticated interfertile non-native trouts in this area, and recommend the adoption of Evolutionary Significant Units for salmonid fishery management. We further propose future research directions for a sustainable approach to the conservation and ecosystem management of the fishery resources and inland waters of northern Italy.

## 1. Introduction: The Global Cultural Value of Trout and the “Trout Wars”

In Europe, freshwater angling originated as subsistence and small-scale artisanal fisheries serving local markets, growing alongside sportfishing as early as in the Late Middle Ages, virtually becoming a synonym of trout fishing [1,2,3]. Analogous subsistence trout-fishing cultures also developed in hunting and gatherer societies, such as the Native American cultures [4].

Sportfishing and trout fly fishing greatly expanded during the 19th century, spreading throughout the world through European colonialism (America, Asia, Africa, Australia, and New Zealand) and the rise of the nation-states, associating with cultural, economic, spiritual, and recreational values [3]. Artificial propagation was scientifically described and popularized in France in the 1840s–1850s. In the European imperialistic societies, highly interconnected by steamships and railroads, these propagation methods fueled a burgeoning outdoor recreation industry, i.e., the consumption of nature for leisure-class consumers [5]. This sporting culture gave rise to present-day fishery management and angling, making trout a globalized commodity through the introduction, acclimatization, and naturalization of non-native trout species provided by private and governmental associations. The European diaspora to other continents following World War I further accelerated this process. “Salmonizing” became a synonym of acclimatization [6]. The first Italian hatchery was established in Piedmont in 1859 [7], and experiments of artificial propagation and acclimatization of non-native fishes, namely *Coregonus* and *Salvelinus* species from Switzerland and Germany, were conducted in several subalpine lakes during the 1860s–1890s [8]. Introductions and translocations accelerated with the establishment of the two Italian fishery centers in Brescia and Rome, in 1893–1895, with jurisdiction over the Padano-Venetian ichthyographic district and the rest of peninsular plus insular Italy, respectively [9,10]. Massive acclimatization and translocation activities (~12 billion fishes of different species, including 16 non-native species) were rapidly implemented in the following 50 years, slowing down after 1945 [10].

During the 20th century, the political impetus to meet the growing anglers’ demand for more fish by turning recreational fishing into a profitable economic sector further promoted the fish-culture industry, which increasingly focused on enhancing both native and non-native fish populations by stocking. The synergistic effects of massive stocking and the habitat degradation associated with the ongoing urbanization increasingly impacted freshwater systems, eventually triggering dramatic conservation and management issues. Artificially stocking more manufactured fish than the ecosystems’ carrying capacity and redefining nature as a play garden temporarily allowed to ignore the ongoing damage to the ecosystems [3,11]. In particular, massive stocking of non-native trouts started to severely impact native assemblages, both ecologically and genetically, due to the widespread interfertility among salmonid taxa, e.g., [12].

In the 1960s, after World War II, the modern ecological movement and the concepts of wild trout management and ethics gradually developed in the U.S.A. alongside put-and-take fishery practices, initially focusing on pollution, habitat degradation and fragmentation, and, then, extending to fishing regulations and restoration of wild native populations. Harsh confrontations between conservationists and advocates of stocking sparked everywhere, from U.S.A. to South Africa, being described as the “trout wars” [3].

In Italy, stocking activities dramatically increased in the 1970s, after the management of inland waters passed to the Provinces in 1974 [9,10]. For at least the subsequent 25 years, stocking was conducted by few large aquaculture facilities. The largest one, located in the Veneto Region, was widely used by several northern and central Italian Provinces to stock massive amounts of several poorly-determined fish species from the Padano-Venetian ichthyographic province, vaguely defined “pesce bianco” (literally: “white fish” [9]). More recently, government compliance with European legislation [13] confirmed the prohibition to stock non-native fish species and populations into Italian waters, unless a site-specific environmental impact assessment demonstrates the lack of negative impacts on native species and the environment [14,15]. The government proposed a reference list of Italian native and non-native species of interest in the fisheries sector. Among the non-native fishes there is one of the world’s worst invasive species, the Atlantic brown trout *S. trutta* Linnaeus 1758 [16], which has been the backbone of the stocking and sportfishing Italian industries for almost two centuries. As a consequence of the strong social and political tension, a recent amendment to the national Budget law [17] has been approved [18] to suspend the application of these laws [14,15] until 2023. A harsh conflict is taking place between stakeholders prioritizing the genetic and ecological diversity of native populations, mainly including fishery scientists plus some anglers and managers, and those prioritizing the exploitation of inland fisheries, mainly including fish culturist, anglers, sportfishing associations, the hydropower private sector, and the majority of the Province and Region administrations. This clash was particularly dramatic in the industrialized north of Italy, home to the Italian Alpine and subalpine trout populations, and source of most of the revenues of the national stocking and angling industries. This “Italian trout war” also provoked heated debates on the taxonomic and genetic identity of the stocked trouts, e.g., [19], and on the native status of trout species in Italy, e.g., [20,21,22,23]. Since 5–10 years, several Italian fish-culture companies started to introduce massive amounts of non-native stocks of peninsular trout (Section 2) in this and other geographic areas. Such stocks are collected from several locations in peninsular and insular Italy, including domesticated progeny often hybridized with non-native Atlantic stocks [19]. These are being sold as “Mediterranean trout”, e.g., [24], and currently advertised as a conservation-friendly alternative to the non-native Atlantic brown trout. This commerce has also been facilitated by the idea, arguably unsupported by any scientific evidence, of a widespread presence of viable populations of a peninsular trout lineage in northern Italy (i.e., *S. cenerinus*; Section 2 and Section 4).

On the other hand, the current global interest in trouts, facilitated by social platforms and Internet virtual communities, initiated several environmental ethics and angler-driven conservation initiatives (e.g., Trout Unlimited in the U.S.A.; Balkan Trout Restoration Group in Slovenia; numerous European LIFE projects). Shared attachment to place, characterizing a wide range of fish enthusiasts, from anglers to fishery scientists [3], could raise scientific awareness and foster collaboration among different stakeholders. This could develop global networks of multiple parties addressing issues such as sustainability, wild management, and transboundary conservation issues, such as climate change, pollution, and obstacles to fish movements (e.g., dams and hydroelectric power plants).

We review here the state of art of the taxonomy and evolution of the Italian trouts, clarifying our scientific view of these topics, and proposing future directions for a scientific approach to the conservation and ecosystem management of the fishery resources and inland waters of northern Italy.

## 2. Native Italian Trouts and the Taxonomy of the “Peninsular Trout”

Among the valid nominal taxa [25] of the native trouts described in the Italian peninsula and the major Italian islands, *Salmo cettii* Rafinesque-Schmaltz 1810 was described from Sicily (type locality: Val Demone in northeastern Sicily and Val di Noto in southeastern Sicily, no types known [25,26]). *S. marmoratus* (Cuvier 1829) is a subendemism of northern Italy described from the “lacs de Lombardie” (syntypes not available [25,27]; Section 3). *S. cenerinus* Nardo 1847 was described from northeastern Italy (type locality: not far from the sea, in rivers draining to the Venetian lagoon; no types known [25]). The original description of *S. cenerinus* was written from the late 1700s to the early 1800s by S. Chiereghin, and published posthumously [28]; a summary of this description was first published by Nardo [29]. *S. macrostigma* (Duméril 1858) has been considered by several authors as an Italian trout; however, it was described from North Africa (type locality: Oued-el-Abaïch, Kabylie, Algeria [25,30]). *S. ghigii* Pomini 1941 was described from central Italy (type locality: Sagittario River; no types known [25,31]). *S. fibreni* Zerunian and Gandolfi 1990, described from the Lake Posta Fibreno in central Italy, and *S. carpio* Linnaeus 1758, described from Lake Garda, are restricted endemisms defined by ecomorphological and genetic traits [32]. The island of Sardinia might host an undescribed *Salmo* species [12].

The short description and illustration of *S. cenerinus* [29] may correspond to the pelagic morph of several anadromous *Salmo* species ([33,34], pers. obs.). Nardo [35] modified his previous view [29], raising doubts on the original description of *S. cenerinus*, accepting the view of [36] (cited as 1858 by [35]), and eventually considering this taxon as a synonym of *Trutta fario* L. (= *S. trutta*). Heckel and Kner [36] reported only two trout species from the Venetian Provinces: *Salar Ausonii* Valenciennes 1848 [37] (= *Trutta fario* L. sensu [35]) and *Fario carpio* (= *Trutta carpio* sensu [35] = *S. carpio*) from the Garda Lake. While *Salar genivittatus* Hecker and Kner 1858 was subsequently recognized as a morph of *S. marmoratus* [38], Heckel and Kner [36] considered *S. marmoratus* as a color morph of *Salar Ausonii*. Therefore, Nardo [35] likely considered the marble trout of this area as color morphs of *Trutta fario*.

Kottelat [38] assigned *S. cettii* to the native peninsular Tyrrhenian and southern Italian trout, including islands, and “tentatively” assigned *S. cenerinus* to the native north-Italian (Adriatic) peninsular trout. Consistently, he did not consider *S. cenerinus* as jun. syn. of *S. marmoratus*, since “there would be no available name for the present species and it should be either listed as *Salmo* sp. or a new name should be created for it”. Kottelat [38] also synonymized *S. ghigii* with *S. cettii* apparently only because Pomini [31] was unable to discriminate the trouts of the Sagittario River from the Sardinian trouts. Kottelat and Freyhof [39] accepted the point of view of Kottelat [38], while noting that “recent studies (…) suggest that the trouts of Sicily (…) belong to a distinct molecular lineage (…). If confirmed, this lineage should retain the name *S. cettii*; the name *S. ghigii* would probably be the valid name of the others”.

Using combined mitochondrial (mtDNA) and nuclear (nDNA) markers, Segherloo et al. [12] found a close relationship between the Sicilian trout of Val di Noto and Atlantic *S. trutta*. A consistent result was found by another nuclear phylogenetic study of the Moroccan trouts, which included the Sicilian trout of Val di Noto in a robust “Afro-Atlantic clade”, likely originated from a colonization wave of an Atlantic lineage from Iberia (“Duero” lineage; [40]). The only North-African sample analyzed by Segherloo et al. [12], that these authors tentatively assigned to *Salmo pellegrini* Werner 1931, is closely related to Mediterranean and Adriatic brown trouts, thus clearly belonging to a different lineage; this sample comes from the Oum er-Rbia River, where Snoj et al. [40] identified trouts of an “Atlas clade”. Several studies showed that the Sicilian trout is morphologically distinct from other Italian trouts [30,41,42,43,44]. Mitochondrial phylogenies also show that the Sicilian trout is included in a clade of North-African trouts, which also includes the sequenced types of *S. macrostigma* [45] and the Atlantic trout lineage, called the “Southern Atlantic clade” [22,46]. However, no nuclear or combined mitochondrial and nuclear phylogenies were ever constructed including the types of *S. macrostigma*, which may be unrelated to the Afro-Atlantic clade.

**Figure 1:**
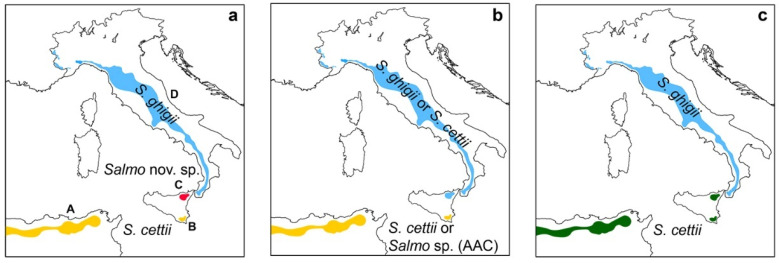
Three possible taxonomic scenarios (peninsular and Sicilian trouts, plus a North-African trout) if native trout samples are collected and analyzed from the Val Demone (no material presently available); (**a**) the native Val Demone population belongs to an undescribed and endemic lineage; (**b**) the native Val Demone population is conspecific with the peninsular trout; (**c**) the Val Demone and Val di Noto populations are conspecific. A: hypothetical distribution of a trout taxon of the “Afro-Atlantic clade” (AAC), conspecific with the Val di Noto trout population [40]; B and C: Val di Noto and Val Demone (likely extirpated [47]) populations, respectively, both described as *S. cettii* by Rafinesque-Schmalz [26]; D: peninsular trout, distribution range modified from [48]. No types are known for both *S. ghigii* and *S. cettii*. The taxon *S. cettii* is older than all North-African trout taxa [48].

Rafinesque-Schmaltz [26] described *S. cettii* from two trout populations: Val di Noto and Val Demone. The molecular phylogeny of *S. cettii* has been investigated only using the former population, since no genetic samples have ever been collected and analyzed from the Val Demone, which has likely been extirpated [47]. The recovery and analysis of any such molecular sample (e.g., from a museum lot) would have important consequences on the scientific names of Italian trout lineages. There are three possible scenarios: (i) the Val Demone population belongs to an undescribed endemic trout lineage; (ii) the Val Demone population is conspecific with the peninsular trout; (iii) the Val Demone and Val di Noto populations are conspecific (Figure 1).

In the first scenario (Figure 1a), the Val di Noto and the North-African Afro-Atlantic clade (AAC, sensu [40]) would be classified as *S. cettii* (older than any North-African trout taxon so far described [48]), the peninsular populations as *S. ghigii*, and the Val Demone population would belong to a undescribed species that would require formal description. Also in the second scenario (Figure 1b), the collection of Rafinesque-Schmaltz [26] contained two distinct taxa, with the Val Demone population being conspecific with the peninsular trout. In the absence of type material, it is arbitrary whether to assign either of the two sampled populations to *S. cettii*. Therefore, there are two possibilities: either (a) Val di Noto plus AAC populations could be classified as *S. cettii*, and Val Demone plus peninsular populations as *S. ghigii*, or (b) Val Demone plus peninsular populations could be classified as *S. cettii*, and Val di Noto plus AAC as one of the four North-African *Salmo* species (including *S. macrostigma* [48]), or as a new and yet undescribed species. In the third scenario (Figure 1c), the Sicilian trout plus AAC would be *S. cettii*, and the peninsular populations would be *S. ghigii*. In the absence of material from the Val Demone, we adopt a classification consistent with the third and most parsimonious scenario.

With the limitation of substantial sample biases, several studies did not find genetic or ecological discontinuities between native northern (South-western Alps [49]) and central-southern peninsular trout lineages that would justify the designation of different taxa, except *S. carpio* and *S. fibreni* [20,32,50,51,52,53,54]. Segherloo et al. [12] assigned trout samples of the upper reaches of the Po drainage to *S.* cf. *cenerinus*, and samples of the Zrmanja and Mornos basins (Balkan peninsula) to *Salmo farioides* Karaman 1938, in the same area of its type locality (Krka River, Croatia [55]; neotype designated by Bianco [56]). *S. cenerinus* was found in brackish conditions [28,29], however the only native Italian trout recorded in the sea is *S. marmoratus* [57]. Further, there presently are no known native populations (nor genetic traces of past populations) of peninsular trout in the area where *S. cenerinus* was described. On the other hand, anadromous non-native populations of *S. trutta*, including hybrids, are known to occur in the Adriatic region, including Italian waters [58,59]. Chiereghin died in 1820, and likely described *S. cenerinus* from the late 1700s to the early 1800s. Fish culture projects, possibly including non-native *S. trutta*, started in this area in the second half of the 19th century [35]. On the other hand, the hypothesis that the trout described by Chiereghin was a pelagic morph of *S. trutta* cannot be ruled out, since introductions of non-native salmonids, possibly including *S. trutta*, have occurred in northern Italy at earlier times (Section 4). In the 1970s, Borroni and Grimaldi [8] just reported that introductions of non-native *S. trutta* had been occurring “for decades” in Italy. Bianco and Delmastro [60] and Bianco [56] synonymized *S. marmoratus* and *S. cenerinus* based on the illustration of *S. cenerinus* [28], its anadromous habits [28,29], and information gleaned from Gridelli [61], who reported only the presence of the marble trout in the Venezia Giulia Region, previous to stocking activities of non-native brown trouts, which started in 1934. However, Nardo’s [29] Venetian Provinces of the 1850s (type locality of *S. cenerinus*) are geographically distinct from Gridelli’s [61] Venezia Giulia Region of the 1930s [62]. Bianco [56] synonymized *S. ghigii* with *S. farioides*, however: (i) no neotype of *S. ghigii* was designated, likely due to the difficulty of finding “purebred” individuals in the type locality; (ii) no molecular analyses were conducted; (iii) the synonymy was essentially based on coloration patterns and biogeographical reconstructions. Therefore, we choose not to consider *S. ghigii* as a junior synonym of *S. farioides*. As a result, until evidence is provided of introductions of non-native *S. trutta* in the area where *S. cenerinus* was described, we consider *S. cenerinus* as jun. syn. of *S. marmoratus* sensu Bianco [56]. While genetic differences between peninsular-trout populations have been found at different geographic scales [23,63], until more comprehensive genetic and ecological data are made available on Tyrrhenian and Adriatic native Italian trouts, we tentatively consider *S. ghigii* as a valid name for all the populations of Italian peninsular trout, sensu Zanetti et al. [64] and Lorenzoni et al. [65].

## 3. Phylogeny and Phylogeography of *S. marmoratus*

Nuclear phylogenetic reconstructions and molecular clocks defined a robust *S. marmoratus* clade, including two distinct northern and southern Adriatic clades that diverged ~0.84 ± 0.4 million years ago (mya) [66,67], and whose taxonomic status has not yet been evaluated. Pustovrh et al. [67] showed that *S. marmoratus* is closely related to a nuclear “*S. trutta* complex” lineage, including several clades associated with different brown-trout taxa, and estimated the divergence between these two lineages at 1.4 ± 0.8 mya (2.2–0.6 mya). A fossil-calibrated nDNA phylogeny estimated an earlier divergence, at ~4–5 mya [68]. An extensive molecular phylogeny combining nDNA and mtDNA sequences rooted with *S. salar*, essentially consistent with previous nDNA phylogenetic reconstructions, supported *S. marmoratus* as a phylogenetic species of possibly hybrid origins, sister to a clade including >20 *Salmo* species [12].

In northern Italy, northern Adriatic *S. marmoratus* populations are strongly associated with the “Marmoratus” (MA) haplogroup of the mtDNA control region (D-loop) [22,69,70,71,72]. However, MA haplotypes have also been found in several brown trout taxa and populations of Greece, Albania, Croatia, central Italy, and Corsica, e.g., [23,48,66,71]. Like several other brown trout taxa and populations, in the Balkans Southern Adriatic *S. marmoratus* populations can be associated with the “Adriatic” (AD) mtDNA haplogroup [67,72].

Mitochondrial molecular clocks estimated much more recent origins of the MA and AD haplogroups (0.21–0.05 mya and 0.39–0.13 mya, respectively, considering the 95% highest probability density intervals estimated using two different substitution rates [23]) than the time of divergence between *S. marmoratus* and the nuclear “*S. trutta* complex”. It was suggested that the observed mitochondrial-nuclear phylogenetic discordance might be the effect of incomplete lineage sorting or asymmetric introgressive hybridization (mtDNA capture; e.g., [73]). The much older time of divergence between these lineages relative to the time of haplogroup differentiation strongly supports the latter mechanism. Paleointrogressive hybridization between the marble trout and the Apennine Mediterranean trout could have occurred during several secondary contacts as a consequence of the expansion of the Po paleo-basin during glacial maxima, as it occurred in other *Salmo* species [22,32,53,59,66,74]. Mosaic distributions of mtDNA haplogroups among different taxa are common also in areas without a history of non-native trout’s stocking (e.g., Albania [75]), and similar distributional and diversity patterns might have occurred in Italy after the Last Glacial Maximum (LGM ~18,000 years ago).

Phylogenetic patterns, molecular clocks and the zoogeography of congeners suggest that *S. marmoratus* is one of the *Salmo* lineages that diverged in the paleo-Adriatic drainage, in freshwater refuges formed during the preceding Lago Mare phase (~5 mya). During the Pleistocene, reduced salinity, cooler sea temperatures, and extensive palaeo-river basins would have facilitated the westward dispersal of these freshwater lineages across the region through multiple waves of colonization, bottlenecks, and secondary contacts [32,72,75], allowing *S. marmoratus* to colonize the orographic left tributaries of the palaeo-Po basin [56,76,77]. After the LGM, increased salinity levels and sea-level rise disconnected these populations, facilitating allopatric fragmentation and differentiation of mtDNA lineages, resulting in the present geographic distribution [32].

In the northern Adriatic basin, *S. marmoratus* exhibit a west-to-east geographic gradient in MA-s1 and MA-s2 haplotype distribution, consistent with the described stepwise westward migration and phylogeographic scenario [78]. Significant microgeographic genetic differentiation was also measured within basins, e.g., between rivers and their tributaries, suggesting the presence of limited gene flow among different populations [79,80]. A contact zone between *S. marmoratus* and *S. ghigii* was found in the South-western Alps (Section 4).

## 4. Presence of *S. ghigii* in the Italian Alpine and Subalpine Region

Within the Italian Alpine region [81], viable native populations of *S. ghigii* (Section 2) have only been found in the South-western Alps (Cottian and Maritime Alps: upper Stura di Lanzo, upper Dora Riparia, upper Chisone, upper Pellice, upper Po, upper Stura Demonte, upper Gesso, and upper Tanaro basins), where a contact zone with *S. marmoratus* was described, along a geographic distribution gradient of genetic variants associated with different trout phyletic lineages [20,21,22,23,34,48,50,77,80,82,83]; Section 3. The MA, AD, and “Mediterranean” (ME) haplotype probability densities relative to elevation show an altitudinal zonation suggesting local habitat differentiation between the two parapatric species, with *S. marmoratus* being dominant at 0–1000 m above sea level (asl) and *S. ghigii* at 1000–2000 m asl [23]. These findings are consistent with the distributional patterns of trouts with different phenotype, described in some historical accounts [84,85,86].

The South-western Alps are a known glacial refuge, where native populations of *S. marmoratus* and *S. ghigii* could have survived the LGM [82]. Introgression rates of alien Atlantic genes into native trout populations are here highly variable (0–70%; [20]). In contrast, in most of the North-western and South-eastern Alps [81] only the lower tracts of the rivers were unaffected by the ice cap during the LGM. Assuming that *S. ghigii* and *S. marmoratus* exhibited a habitat segregation pattern analogous to that presently observed in the South-western Alps, the LGM likely allowed the survival of *S. marmoratus* at lower altitudes, while *S. ghigii* might have been pushed into the marble trout habitat and outcompeted [82]. After the last glaciation, most Alpine lakes and headwaters may have only marginally been colonized by *S. marmoratus* and likely remained “fishless” (i.e., troutless). In historical times, these systems were artificially stocked with translocated salmonids, including non-native Atlantic *S. trutta* [82], to support subsistence and recreational fisheries [87,88,89]. The capacity of *S. marmoratus* to outcompete other trout species was observed by Sommani [90], who observed that in specific water courses marble trout is able to rapidly replace brown trout (*S. trutta fario* = *S. ghigii* or *S. trutta*; this author was unable to discriminate between these species), when restocking practices are interrupted.

In the Lake of Garda basin, a known glacial refugium [91] in the South-eastern Alps [81], a study [92] found traces of the mitochondrial variant ADcs-1 (the most widespread AD haplotype [23], typically associated with the “Adriatic grouping” of *S. trutta fario*, sensu [50] = *S. ghigii*). The prehistoric presence of *S. ghigii* in the Lake Garda refugium is also consistent with the presence in *S. carpio* of haplotypes phyletically related to haplogroups typically associated with *S. ghigii* (AD) and *S. marmoratus* (MA), suggesting that one or more paleohybridization events occurred in this basin between these trout lineages [20,23,50,53]. This also supports the hypothesis of extensive secondary contacts and hybridization events between peninsular and marble trout lineages before the last glaciation in this region [53]. The ADcs-1 haplotype was also found in two museum specimens with lacustrine morphs collected in Lake Garda and Lake Maggiore in 1877 and 1879, respectively [83]. Lake Maggiore is another known glacial refugium [91,93] located in the North-western Alps [81]. The presence of AD haplotypes in these basins suggests that relict populations of *S. ghigii* might have survived the LGM in other glacial refugia of the North-western and South-eastern Alps. More speculatively, since *S. marmoratus* is the only native trout with lacustrine morphs in this region, this might also indicate the more common presence of marble trouts with AD haplotypes in this basin in past historical times, or even the presence of recently extinct and undescribed trout taxa [20,94,95].

With the only exception of the South-western Alps, the absence of viable populations of *S. ghigii* in northern Italy clearly indicates that all relict populations of *S. ghigii* that might have survived to the LGM in other glacial refugia were subsequently extirpated. This might have reasonably occurred due to demographic or genetic swamping [96] caused by the man-made massive and prolonged introductions of non-native *S. trutta* in historical times. Consistently, introgression rates of Atlantic *S. trutta* into *S. marmoratus* are higher in the North-western and South-eastern Alps, and only traces of the haplogroups typically associated with native *S. ghigii* were found [21,82]. This scenario is supported by the probabilistic approach adopted by [92], which showed that, in spite of the massive introductions of *S. trutta*, genetic traces of extirpated *S. ghigii* populations could still be found in some glacial refugial areas such as the Lake Garda basin. Such dramatic effects could have been facilitated by strong numerical differences between native *S. ghigii* populations and *S. trutta* introductions, low hybrid fitness, and weak reproductive barriers. By contrast, the presence of partial reproductive barriers between non-native *S. trutta* and *S. marmoratus* [80,97], the competitive advantage of *S. marmoratus* [90], and marble-trout stocking could have prevented the lineage or local genomic extinction of the latter species. In spite of the presence of high introgression rates [82], neither demographic swamping nor local genomic extinctions of native Apennine *S. ghigii* have ever been described in the Tuscano-Latium Italian ichthyogeographic region, where non-native *S. trutta* have been and are being introduced. On the other hand, these *S. ghigii* populations were much less impacted by habitat modification or competition with other species during the LGM, and were likely larger and less fragmented when they were flooded by *S. trutta* introductions.

Some studies found the allozymic variants LDH-C1*100 and TF*102, typically associated to *S. ghigii* populations in trout populations native to France and south-west Piedmont, in sites collected east of the South-western Alps, hence suggesting the presence of *S. ghigii* outside the mentioned contact zone with *S. marmoratus* [23]. However, these allozymes were also found at high frequency in Danubian native populations of different *Salmo* species [49,98,99]. In one of these studies, Largiadèr and Scholl [100] assumed the native status of an “Adriatic fario” in a large portion of the Po basin, based on molecular studies conducted in south-western Piedmont [49] and on phenotypic studies that were however unable to discriminate between Atlantic (*S. trutta*) and Adriatic (*S. ghigii*) trout phenotypes [101]. These authors found these two allozymic variants at high frequency (~20–30%) in Engadin (Danubian basin), in the Müstair, tributary of the Adige River, and in the Poschiavo valley (Po basin, Poschiavino Torrent, tributary of the Adda River); and at low frequency (~0–10%) in the Ticino and Valais basins, including a tributary of the Diveria Torrent (Chrummbach). No “purebred” individuals were found. In fact, all these populations had been directly or indirectly either entirely replaced or heavily stocked with trout lineages of the Danubian basin via the Poschiavo hatcheries, for at least one century before the study collection [100]. This would explain the genetic similarities between the trouts of the Poschiavino and Ticino valleys, subsequently detected by other studies using microsatellite and AFLP markers to investigate the adaptive divergence and phylogeographic patterns of trout populations of the Rhine, Rhone, and Po basins [102,103]. Just like [100], also these studies assumed the presence of an “Adriatic trout” (*S. cenerinus*, sensu [39] = *S. ghigii*; Section 2) in the Poschiavino and Ticino valleys, based on the literature [39,100,101]. However, given the lack of genetic references (allele size range) of Danubian trout populations (possibly *Salmo labrax* Pallas 1814 [102,103]) it is not possible to know whether the observed “Adriatic” genetic traces in the Poschiavino and Ticino valleys were originally present in this region, or were left by introduced Danubian stocks [102], as also suggested by the presence of Danubian mtDNA haplotypes (DA haplogroup) in the Ticino basin [78]. Keller et al. [102,103] also found evidence of introgression of the Poschiavo population into one Rhine population (SE). SE is the closest Rhine population to the Danubian drainage, suggesting the presence of stocking activities and translocations between SE, Poschiavo, and Danubian systems.

There are several descriptive accounts (cuisine recipes, anecdotes, poetry, and even paintings e.g., [104]) of trouts in the North-western and South-eastern Alps (e.g., Lakes of Como and Garda basins) before the dramatic expansion of the fish-culture industry that promoted the rapid diffusion of the non-native Atlantic *S. trutta* in the early 19th century (1850–1893, [10]). Several ones [105,106,107,108,109,110,111,112] depict or describe trouts without a marbled coloration pattern and with either red and black dots, phenotypically compatible with several trout taxa, or with speckled dark patterns on a silvery background, compatible with a generalized pelagic (lacustrine) morph of anadromous trout. Adult *S. marmoratus* living in rivers typically exhibit a marbled coloration pattern [113]; however, anadromous individuals in pelagic conditions can exhibit a silvery and dark-speckled coloration pattern, even leading to taxonomic confusion, e.g., [114].

**Figure 2:**
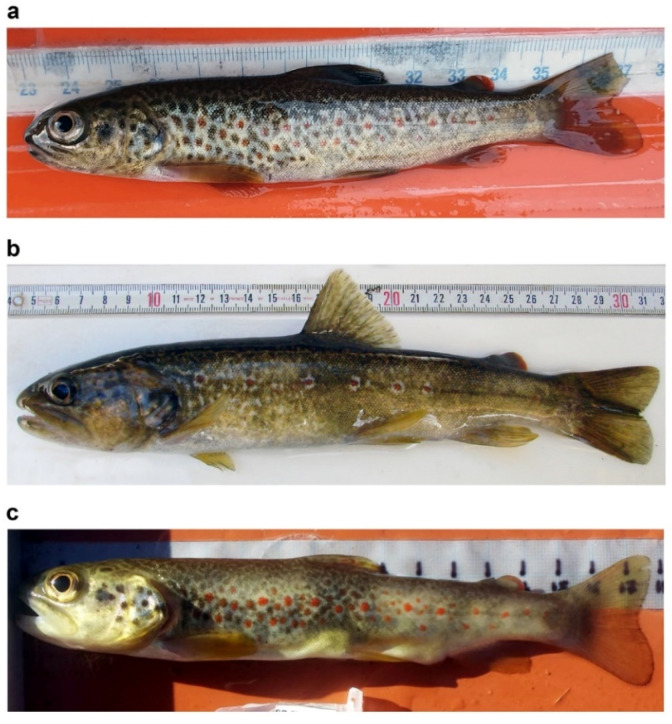
Examples of dotted coloration patterns in *S. marmoratus*: juvenile coloration pattern (**a**), and adults living in small and fast-flowing streams (**b**,**c**); (**a**) juvenile from Roledo (Piedmont, Verbano-Cusio-Ossola: VCO; 46°10′16.7″ N 8°18′49.7″ E), 15.5 cm total length—TL, 29.0 g wet mass, 22 months of age, black-and-red dotted pattern, MA haplogroup, q_Ma_ 0.995 (admixture proportion of a cluster including purebred *S. marmoratus* references), 90% BCI 0.966–1.000; (**b**) adult (reproductive) specimen from Rio Ischielle, tributary of the Avisio Torrent (Province of Trento); the specimen was collected from a population which resided for 2 generations in this small stream, which descended from hatchery-reared *S. marmoratus* with marbled phenotype collected from the Adige River [115]; 26.9 cm TL, courtesy of Leonardo Pontalti; (**c**) adult (reproductive) specimen from Rio della Balma, tributary of the Sangone River (Province of Torino), 18.5 cm TL, MA haplogroup, q_Ma_ 0.996, 90% BCI 0.978–1.000, courtesy of Paolo Lo Conte.

**Figure 3:**
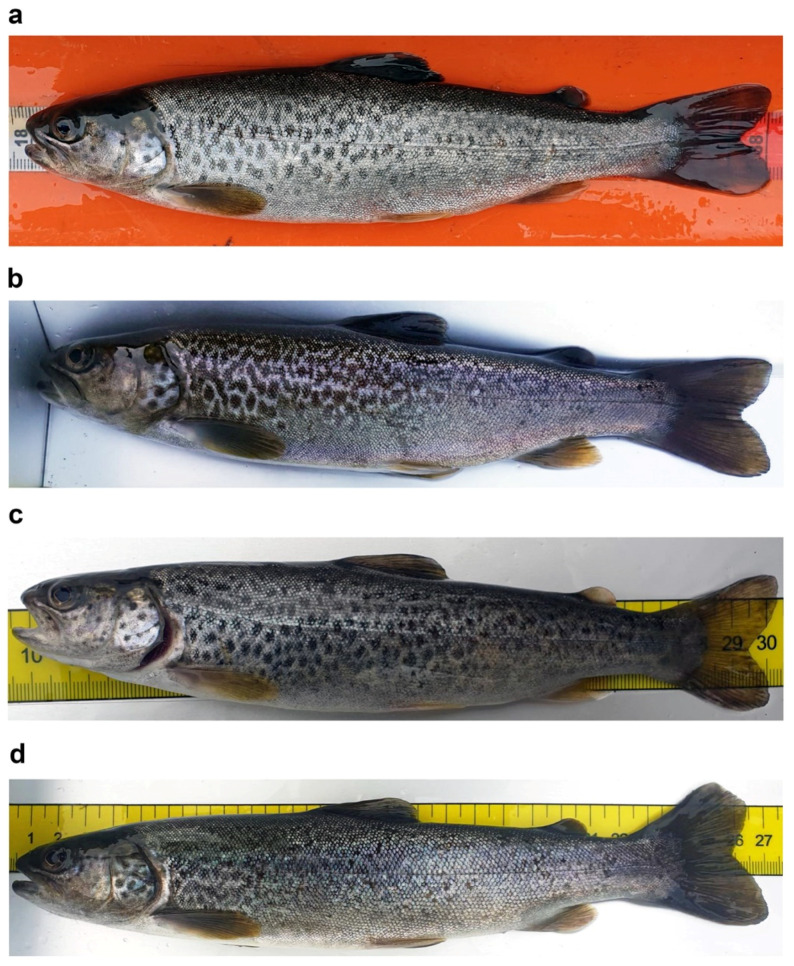
Examples of dotted coloration patterns in *S. marmoratus*: conspicuous ontogenetic chromatic variation in pit-tagged individuals which were recaptured at different times; (**a**,**b**) subadult specimen sampled in Roledo (Piedmont, Verbano-Cusio-Ossola: VCO; 46°10′16.7″ N 8°18′49.7″ E), age and genetic data unavailable; (**a**) sampled on 28 April 2021, 20.0 cm TL, 86 g, dotted pattern; (**b**) recaptured in the same site on 28 October 2021, 23.4 cm TL, 122 g, marbled pattern; (**c**,**d**) Subadult specimen sampled in Prata di Vogogna (Piedmont, VCO; 46°1′40.8″ N 8°17′2.2″ E), age and genetic data unavailable; (**c**) sampled on 26 April 2021, 20.6 cm TL, weight not available, dotted pattern; (**d**) recaptured in the same site on 19 October 2021, 26.4 cm TL, 166.0 g, marbled pattern.

Young marble trout typically exhibit an irregular black or red-and-black dotted pattern, with a large black preopercular blotch, similar to adult brown trouts [113] (Figure 2a); the dark dotted pattern can change to a marbled pattern in a few months in subadults (Figure 3a–d); and adults living in small and fast-flow streams can become reproductive at half the typical length at maturity, while retaining a “brown-trout” red or red-and-black dotted pattern [115] (Figure 2b,c).

On the other hand, there is ample evidence of salmonid introductions in old historical times from outside Italy. Domestication practices and translocations of freshwater fishes, even across mountain ranges, go back to the Middle Ages and possibly to the Neolithic, seamlessly continuing through to the 18th and 19th century, before the onset of the fish-culture industry [10,116,117,118,119]. Non-native trouts with “brown-trout” dotted coloration patterns could have been introduced in northern Italy from adjacent areas such as the orographic right tributaries of the Po River, or even beyond the Alpine Divide, e.g., from the Danube basin, such as the common carp *Cyprinus carpio* L. in the Roman Period [10,120].

**Figure 4:**
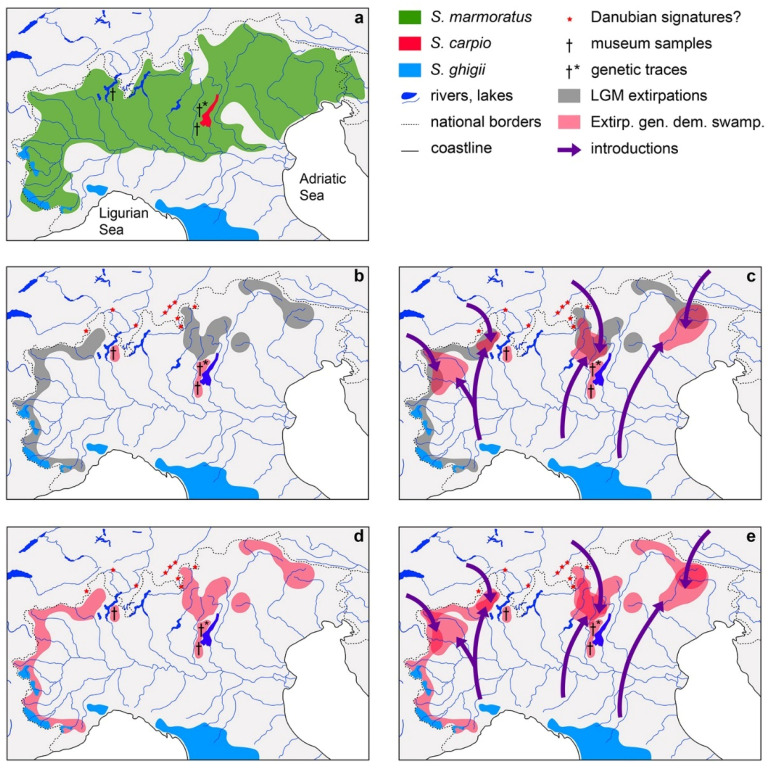
Present distribution of trouts in northern Italy and hypothetical reconstructions of the demographic history of *S. ghigii* in this geographic area; (**a**) present distribution of Alpine and subalpine trouts in northern Italy, modified from [23,48,90,121]; (**b**) graphical representation of the hypothetical distribution of *S. ghigii* that were extirpated during the LGM except in glacial refugia, lack of subsequent introductions of *S. ghigii* in historical times, and subsequent extirpations of relict native populations in glacial refugia (e.g., Lake Maggiore, Lake Garda, except the South-western Alps), due to genetic or demographic swamping caused by the massive introductions of *S. trutta* during the last ~150 years; (**c**) as in (**b**), but with man-made introductions of *S. ghigii* from areas outside its original distribution; (**d**) hypothetical distribution of *S. ghigii* populations that survived the LGM, lack of subsequent introductions of *S. ghigii* in historical times, and subsequent extirpations of relict native populations in glacial refugia, except the South-western Alps, due to genetic or demographic swamping caused by the massive introductions of *S. trutta*; (**e**) as in (**d**), but with man-made introductions of *S. ghigii* from areas outside its original distribution. In legend, present distribution of the three trout species: *S. marmoratus* (green area), *S. carpio* (red area), and *S. ghigii* (blue area); Danubian signatures?= records of genetic variants possibly introduced from the Danube basin [100,102,103]; museum samples= records of ADcs-1 in two 19th-century museum samples [83]; genetic traces= potential traces of extirpated *S. ghigii* populations [92]; LGM extirpations= hypothetical distribution (grey area) of *S. ghigii* populations that were extirpated during LGM; Extirp. gen. dem swamp. = hypothetical distribution (pink area) of *S. ghigii* populations that were extirpated by demographic or genetic swamping caused by introductions of *S. trutta* in historical times; introductions= directions (purple arrows) of *S. ghigii* man-made introductions from areas outside its original distribution.

Considering (i) the absence of viable populations of *S. ghigii* in northern Italy, except in the South-western Alps (Figure 4a), and the presence of potential genetic traces of extirpated populations in glacial refugia (Figure 4b–e); (ii) the past widespread presence of *S. ghigii* in this region before LGM, supported by the estimated gene flow occurred for tens of thousands of years between the marble and peninsular lineages [53]; (iii) the hypothetical extirpation of most native populations of *S. ghigii* in this region during LGM [82]; (iv) the possibility of recent extirpations of *S. ghigii* populations due to genetic or demographic swamping caused by massive introductions of non-native *S. trutta* during the last two centuries [92]; (v) the anecdotal accounts potentially reporting the presence of *S. ghigii* in this region in historical times, 4–5 centuries before the 19th-century flourishing of the fish-culture industry [104,105,106,107,108,109,110,111,112]; four potentially falsifiable scenarios can be hypothesized (Figure 4):

1. Native populations of *S. ghigii* have been extirpated in most of the region during the LGM, except in glacial refugia. *S. ghigii* was never subsequently introduced from areas outside its original distribution. Then these relict native populations, e.g., the glacial refugia of Lake Maggiore and Lake Garda, have been extirpated by genetic or demographic swamping, due to the massive introductions of *S. trutta*, except those of the South-western Alps (Figure 4b).
2. Native populations of *S. ghigii* have been extirpated in most of the region during the LGM, except in glacial refugia. *S. ghigii* was subsequently introduced from areas outside its original distribution. Then both the relict native and non-native populations have been extirpated by genetic or demographic swamping, due to the massive introductions of *S. trutta*, except those of the South-western Alps (Figure 4c).
3. Native populations of *S. ghigii* survived the LGM in several areas of the region, including glacial refugia. *S. ghigii* was never subsequently introduced from areas outside its original distribution. Then these native populations have been extirpated by genetic or demographic swamping due to the massive introductions of *S. trutta*, except those of the South-western Alps (Figure 4d).
4. Native populations of *S. ghigii* survived the LGM in several areas of the region, including glacial refugia. *S. ghigii* was subsequently introduced from areas outside its original distribution. Then both the relict native and non-native populations have been extirpated by genetic or demographic swamping, due to the massive introductions of *S. trutta*, except those of the South-western Alps (Figure 4e).

In every scenario, *S. ghigii* has been extirpated in this region except in the South-western Alps, and no other native and viable populations are left at present. The populations of *S. ghigii* of the South-western Alps are the only autochthonous ones, therefore being the only ones that might be managed.

In both the first and second scenarios (Figure 4b,c) *S. ghigii* became non-native in the Alpine and subalpine Italian region after the LGM, except in glacial refugia, as indicated by the available scientific evidence. Due to the intense climatic and ecological changes that followed the LGM, it would make little conservational and ecological sense to reintroduce species or populations that lived in northern Italy during the Late Pleistocene and were naturally extirpated during the LGM, e.g., such as the leopard *Panthera pardus* L. [122,123]. Except the South-western Alps, populations of *S. ghigii* may have survived in other glacial refugia, before being extirpated by demographic or genetic swamping caused by *S. trutta* introductions in historical times. Even if these reconstructions are supported by further investigations, any reintroduction of *S. ghigii* in such areas (e.g., Lake Maggiore and Lake Garda basins) should take into careful consideration the present environmental conditions, potential interactions within the community, and habitat availability, since these may have become inadequate to host the species since its extirpation. This case is exemplified by the Eurasian beaver (*Castor fiber* L.), whose north-Italian populations survived in glacial refugia during the LGM and subsequently re-expanded in the region, only to be completely extirpated in the 16th–17th century due to anthropogenic environmental changes [124]. In spite of the sporadic sightings of the Eurasian beaver in northern Italy in 2018 and 2020 [125], the lack of available habitat in this region would likely make a reintroduction program meaningless.

In the third and fourth scenarios (Figure 4d,e), native populations of *S. ghigii* would have survived the LGM in several areas within this region, including glacial refugia, and were eventually extirpated by demographic or genetic swamping caused by the introductions of *S. trutta*. These scenarios lack scientific support, since the presence of potential traces of *S. ghigii* outside glacial refugia was never demonstrated. However, even if the past presence of *S. ghigii* in other areas is demonstrated in the future, the same considerations made for scenarios 1 and 2 apply, in case of extirpations occurred in historical times. Careful assessments of environmental impact must be conducted, before considering any reintroductions of *S. ghigii* in such areas, irrespective of its status preceding its extirpation. Therefore in the absence of reliable data, reintroductions of *S. ghigii* should be avoided, invoking the Precautionary Principle [126].

Notwithstanding, massive amounts of non-native individuals of *S. ghigii* sourced from outside this region have been and are being regularly and massively introduced into this region during the last decade. Such stocking activities will obviously hamper any investigation attempting to assess the status of any relict *S. ghigii* populations that may be found in this region in the future. In fact, any new biological variant found in the region that is not present in any of the non-native source populations may still be non-native, i.e., being still undescribed in the source population.

## 5. Current Risks of Stocking Non-Native *S. ghigii* in Northern Italy

The risk of introgressive hybridization between native *S. marmoratus* and non-native stocks of *S. ghigii* is not only suggested by the widespread interfertility between *Salmo* species [12,72]. The same presence of gene-flow between these trout lineages during past secondary contacts before LGM [53] clearly demonstrates the potential for such events. This is particularly the case when native small and fragmented populations are flooded by large amounts of stocked non-native fish [82], in the same ecological conditions that caused the ongoing introgression between *S. marmoratus* and Atlantic *S. trutta* in this region [127].

The ongoing stocking of individuals of *S. ghigii* obtained from non-native populations is also associated with other risks. While hatchery managers introducing *S. ghigii* in this region typically do not publish any genetic screening of the stocks, an independent investigation in a different geographic area showed that a hatchery stock of “Mediterranean trouts” actually contained a mix of both Atlantic haplotypes (44%) and Mediterranean haplotypes (66%), being characterized by a q_AT_ value (admixture proportion of the cluster characterizing two hatchery stocks of non-native Atlantic *S. trutta*) of 0.42–1.00 [19]. Given the exceptional rarity of such independent investigations, such cases are more likely the rule rather than the exception. Introductions of such hybrid stocks pose an even greater risk than introductions of purebred non-native *S. ghigii*, since hybrids can effectively act as a genetic bridge, facilitating hybridization and introgression between reproductively isolated species [128].

The dispute revolving around *S. ghigii* and the “Mediterranean trout” in northern Italy is also related to another hot and current topic, i.e., the management of stream headwaters. Stocking of native *S. marmoratus* generally occurs in what is considered to be its putative vocational habitat, i.e., in middle and lower river reaches (<1500 m asl [90]). On the other hand, current regulations have often prohibited the introduction of non-native species, such as the Atlantic brown trout, that has traditionally been released in these water courses for >150 years (Section 1). This caused a heated debate, fueled by anglers and sportfishing associations, who advocate the use of non-native stocks of *S. ghigii* or *S. trutta* to exploit stream headwaters. Prior to human settlement or stocking, most headwater streams in this region were likely troutless (Section 4). However, while this hypothesis still lacks experimental support, the idea of stocking non-native *S. ghigii* in these environments is associated with substantial environmental risk, being at odds with conservation principles (Section 1 and Section 4). Headwater streams could be stocked with non-native sterilized fishes, e.g., by a process of triploidization, thus preventing hybridization with native species downstream. However, massive fish introductions can significantly impact the structure and functioning of freshwater ecosystems, due to interactions with the native communities and recipient environments, including increased competition, predation, biogenic modification of the environment, and potential spreading of diseases [129,130,131,132,133,134]. Therefore, the sustainability of this management strategy must be considered with extreme caution, carefully assessed, and regularly monitored after implementation.

## 6. Conclusions and Future Directions

At present, robust scientific evidence identifies *S. marmoratus* plus small and fragmented populations of *S. ghigii* in the South-western Alps as the only native and distinct *Salmo* lineages and populations in northern Italy. Introgressive hybridization from non-native *Salmo trutta* into *S. marmoratus* in this region has been repeatedly demonstrated. Given the evidence of past introgression from the peninsular lineage into the marble lineage, genetic introgression from non-native stocks of *S. ghigii* into native *S. marmoratus* may have already occurred. In particular, the presence in non-native introduced stocks of *S. ghigii* of life-history tracts that would prevent or minimize hybridization with *S. marmoratus* cannot and should not be assumed.

Possible genetic traces of *S. ghigii* outside the contact zone are fragmentary, and strongly suggest past extirpations. It is presently unknown whether viable populations of this species were present in historical times, or most of the native populations were extirpated during the LGM.

Regardless of the academic value of taxonomic debates, the high interfertility among many salmonid taxa makes a rational approach to the ecosystem management of salmonid populations extremely challenging. Any introduction of trout individuals originating from non-native populations defined by phylogeographic and genetic criteria, even if conspecific with the recipient population, poses the risk of generating hybrid swarms between non-native stocks and native trout lineages. For this reason, translocated and restocked salmonid populations should always be managed as Evolutionary Significant Units (ESUs; [32,135,136]). Considering the genetic structure of populations at the microgeographic scale (i.e., hydrogeographic basin and sub-basin) makes conservation actions taxonomy-independent, gaining the sorely needed stability for conservation purposes. In the specific case of reintroductions, using the closest available ESU as a source to rebuild an extirpated population still poses the risk of introducing individuals with different life-history traits than those of the original population. For this reason, the potential impacts of reintroductions should always be carefully evaluated, in case prevented, and then monitored, e.g., [137] (Section 4 and Section 5).

Future research could (i) further our knowledge of the genetic structure and microgeographic patterns of *S. marmoratus* populations within the Italian Alpine and subalpine region, thus identifying ESUs for science-based conservation and management; (ii) investigate the past presence of *S. ghigii* populations in this region using ancient DNA, e.g., in archaeological sites [138] or in ancient lake sediments [139]; (iii) investigate the past presence of salmonids in hypothetically troutless headwater streams prior to stocking, e.g., using museum records; (iv) investigate the presence of vertebrate and invertebrate species of evolutionary and conservation interest evolved in hypothetically troutless headwater streams, and their potential interactions with non-native fish candidates for stocking; (v) monitor spatiotemporal dynamics of genetic diversity of trout lineages, both native and non-native, with special attention to the potential onset and development of new hybrid swarms originating from the recent introductions from *S. ghigii* non-native stocks; (vi) investigate the genetic structure, ecology and conservation status of *S. ghigii* native populations (ESUs) in the South-western Alps, designing and implementing dedicated conservation programs, if needed; (vii) evaluate and implement supporting breeding programs for threatened and heavily fished ESUs of *S. marmoratus*, using state-of-the-art methods to genetically select breeders and minimize domestication effects.

Research efforts will however be insufficient to meet the common goal of sustainable ecosystem management [140], if all the stakeholders are not involved in a durable, empathetic, and collective effort. From anglers to sportfishing associations, hydropower sectors, researchers, conservationists, and governmental institutions, everyone is called to protect, conserve, and promote the native north-Italian trouts, hence preserving the natural heritage of our inland waters.

## Data Availability

Not applicable.
